# Students’ Perceived Heat-Health Symptoms Increased with Warmer Classroom Temperatures

**DOI:** 10.3390/ijerph13060566

**Published:** 2016-06-07

**Authors:** Shalin Bidassey-Manilal, Caradee Y. Wright, Jacobus C. Engelbrecht, Patricia N. Albers, Rebecca M. Garland, Mamopeli Matooane

**Affiliations:** 1 Department of Environmental Health, Tshwane University of Technology, Pretoria 0001, South Africa; shalinb@joburg.org.za (S.B.-M.); engelbrechtjc@tut.ac.za (J.C.E.); 2 Environment and Health Research Unit, South African Medical Research Council, 1 Soutpansberg Road, Pretoria 0001, South Africa; patricia.albers@mrc.ac.za; 3 Department of Geography, Geoinformatics and Meteorology, University of Pretoria, Pretoria 0001, South Africa; 4 Natural Resources and the Environment, Council for Scientific and Industrial Research, Pretoria 0001, South Africa; rgarland@csir.co.za; 5 Climatology Research Group, North West University, Potchefstroom 2520, South Africa; 6 Tlhoeko Environmental Consultants, Maseru 100, Lesotho; matooanemr@yahoo.com

**Keywords:** temperature, health, school, South Africa, climate change

## Abstract

Temperatures in Africa are expected to increase by the end of the century. Heat-related health impacts and perceived health symptoms are potentially a problem, especially in public schools with limited resources. Students (*n* = 252) aged ~14–18 years from eight high schools completed an hourly heat-health symptom log over 5 days. Data loggers measured indoor classroom temperatures. A high proportion of students felt tired (97.2%), had low concentration (96.8%) and felt sleepy (94.1%) during at least one hour on any day. There were statistically significant correlations, when controlling for school cluster effect and time of day, between indoor temperatures ≥32 °C and students who felt tired and found it hard to breathe. Consistently higher indoor classroom temperatures were observed in classrooms constructed of prefabricated asbestos sheeting with corrugated iron roof and converted shipping container compared to brick classrooms. Longitudinal studies in multiple seasons and different classroom building types are needed.

## 1. Introduction

Schoolchildren spend a significant portion of their time attending school and being indoors in school classrooms. In South Africa, and likely in other developing countries [1], the majority of government-funded schools rely on natural ventilation for cooling when ambient temperatures are high. Windows and doors may be kept open, and some schools (though likely to be few) may have ceiling fans, but the likelihood of there being air conditioning in government schools is very low. Furthermore, the class size is often relatively large (*i.e*., ~35–40 schoolchildren) leading to overcrowding and an increase of indoor classroom temperature from body heat. These two factors, when combined with high ambient temperatures (especially those temperatures experienced during summer months) can lead to very warm to extremely warm classroom temperatures in schools. High temperatures may impact schoolchildren’s (and teachers’) health, school learning, productivity and performance as well as their general well-being [2]. In this study, the focus was on the perceived heat-related health of schoolchildren.

Exposure to high temperatures has known adverse effects on human health. These effects may be especially dangerous for children whose physiology is still developing and internal coping mechanisms are yet to be fully operationalised [2]. Heat-related health impacts may be mild, such as nausea, headache and dizziness, or extreme, including vomiting, heat stroke and cardiac arrest [3]. Headache, fatigue and “feeling very hot” were the most common symptoms experienced by Cameroonian schoolchildren when indoor classroom air temperature was high (no threshold was defined) [4]. Schoolchildren may be able to adapt to moderate changes in temperature [5], however, this may not be the case when ambient temperatures increase, which is highly likely given the predicted increase in future temperatures in many parts of the world, and especially in Africa where coping or adaptation measures are limited [6].

Climate model projections predict that in a business-as-usual scenario, ambient temperature is likely to increase in southern Africa twice that of the predicted global average increase predicted to be 2 °C by the end of the 21st Century [7]. Thus, ambient outdoor temperatures in this region may be up to 4 °C higher than those temperatures being experienced today, and this may be worse for African urban areas such as the City of Johannesburg (CoJ) [8]. Given this possible future risk of increasing ambient temperature, which will influence indoor classroom temperatures, particularly in classrooms without air conditioning, it is important to understand both the physiological and perceived heat-health risks of high temperature on schoolchildren in this country. Previous studies in other countries have used thermal comfort indices and questionnaires to assess individual perceptions of thermal comfort, including among children [9,10,11,12,13]. The relationship between indoor environmental conditions in general and student achievement is well established; however, the specific role of thermal comfort is less well understood [14]. The ASHRAE 55 Standard defines thermal comfort as a condition of mind which expresses satisfaction with the thermal environment [15]. The World Health Organization recommends a maximum working temperature of 24 °C [16]. Overheating in classrooms is said to occur when the internal air temperature exceeds 28 °C [17]. In the United Kingdom, performance standards for summertime overheating state that the internal air temperature should not exceed 32 °C when the space is occupied [18]. In South Africa, the Occupational Health and Safety Act 85 of 1993 states that employees, including teachers, should be trained to take action to avoid heatstroke, but only prescribes temperature thresholds for minimum temperature exposures [19].

This study focused on indoor classroom temperature and perceived heat-health risks, as has been done elsewhere [20,21] but never before in South Africa, as a simple and immediate first step towards providing information for evidence-based decision-making by teachers, principals and school managers to ensure appropriate school thermal comfort. The aim of this study was to assess schoolchildren’s perceived heat-health symptoms during school hours in the classroom. Two study objectives were defined, namely, (1) to quantify indoor classroom temperatures in a sample of Johannesburg government schools and (2) to understand schoolchildren’s perceived hourly heat-health symptoms in relation to indoor temperature. This is considered to be the first reported study of its kind. We aimed to evaluate the impact of high temperatures on schoolchildren’s perceived heat-health symptoms in Johannesburg where annual average summer ambient temperatures are generally high and are expected to increase significantly by the end of the century.

## 2. Methods

### 2.1. Study Area and Sample

**Figure 1:**
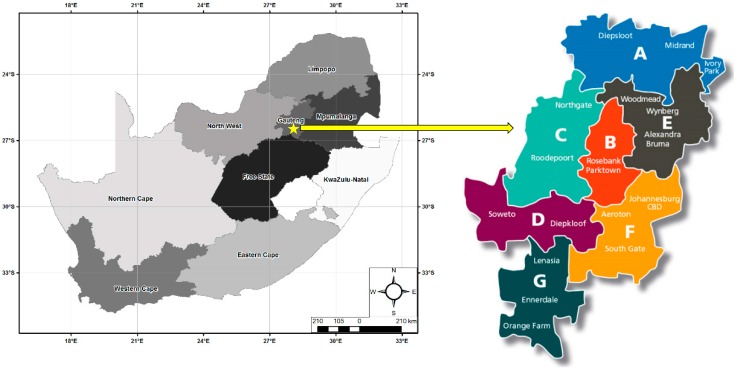
Map of South Africa and the regions within the City of Johannesburg (With permission from The City of Johannesburg, Johannesburg, South Africa).

Data were collected in the CoJ, the largest city in South Africa with more than 8 million inhabitants. The CoJ comprises seven regions of varying socio-economic levels ranging from Region A, C and E in the North, Region B in the centre, to D, F and G in the South. For the purpose of this study, Regions B, F and G were selected for inclusion to represent regions comprising mainly wealthy suburbs Region B), inner city high-rise suburbs of low and middle income groups (Region F), and very low income suburbs dominated by informal dwellings and shacks (Region G) (Figure 1). In the CoJ, average summer temperatures range between 15 °C and 29 °C [8]. 

There are 201 government secondary and high schools in the CoJ. The 2011 census school attendance rate (students aged 5 to 17 years) in the CoJ as a whole was ~89.4% [22]. Constrained by budget and logistical feasibility, a total of eight schools were randomly selected from the three Regions for inclusion in the study. At each school, one class from any grade (8–12) was selected to participate in the study based on principal and teacher willingness. Research ethics clearance was granted by the Tshwane University of Technology Research Ethics Committee (REC2013/11/007) and the Council for Scientific and Industrial Research Ethics Committee (53/2012). Permissions were obtained from the Gauteng Department of Education and the Department of Health, school principals and chairpersons of the school governing bodies. Parents of participating students gave their informed consent and students gave their assent prior to participating in the study. All instruments and procedures were piloted and pre-tested prior to the main study. Schools where students were First or Second Language English proficient were included in the study as all instruments were in English.

### 2.2. Procedures for Data Collection

Panel data were collected. A researcher contacted the school principal via telephone to obtain permission to conduct the study at their school. When the principal (and governing board chairperson, if necessary) agreed, the researcher visited the school to meet with the principal and class teacher who was willing to assist with implementing the study protocol in their class. Informed consent forms were provided to the students to obtain parent/guardian consent. Study dates for the school were agreed upon (see Table 1, row 6) at which time the researcher returned to the school to install the temperature loggers, hand out the student questionnaires and the principal questionnaire. The study ran for a period of 5 days, Monday to Friday, at each school during summer/early autumn. The teacher prompted the students to complete the questionnaire on a daily basis during the course of the school day from 8 h to 14 h. After the 5-day period, the researcher returned to the school to collect the temperature loggers, student and principal questionnaires for processing and analysis.

### 2.3. Temperature and Humidity Measurements

Lascar temperature data loggers were used to measure indoor classroom temperatures and humidity at each school. The logger came with a software configuration as well as a calibration certificate. The logger was mounted from the ceiling in the centre of the classroom at a height about the students’ heads and away from windows and direct sunlight. Loggers were set to record every 5 min and activated on continuous mode. The twelve 5-min interval readings were averaged to obtain an hourly average temperature value that corresponded with the student perceived health symptoms per hour recorded in the questionnaire (described below). Humidity data were used for descriptive purposes only. For comparison, ambient temperature and humidity data (minimum, maximum and mean) for the study days available online [23] were retrieved from the Oliver R. Tambo International Airport in Johannesburg against the classroom indoor temperature and humidity readings at each school. Schools ranged in distance from the airport between 18 km and 38 km. Ambient mean, minimum and maximum temperature and humidity data are provided in supplementary data tables (Tables S1–S3).

In addition to measured temperature, we also calculated apparent temperature using indoor temperature and humidity measurements made in the classrooms. Apparent temperature is an indicator of thermal sensation in indoor settings [24] and has been used before when considering the relationship between heat and health [3]. Apparent temperature was calculated using the 5-min temperature and humidity measurements made by the data loggers and using Equation (1):

AT = Ta + 0.33 × e − 0.70 × ws − 4.00
(1)

where AT is apparent temperature, Ta is measured dry bulb temperature (°C) in the classroom, e is water vapour pressure (hPa) and ws is wind speed [24]. Given that this is an indoor setting, ws was set to 0. Water vapour pressure was calculated using the humidity measurements made in the classrooms and applying Equation (2) as follows:

e = rh/100 × 6.105 × exp (17.27 × Ta/(237.7 + Ta))
(2)

where rh is relative humidity (%). The apparent temperature calculations for the study days were made for comparison and discussion purposes in relation to the measured indoor classroom temperatures. The apparent temperature data were not applied in the statistical modelling of student health symptoms and indoor temperature since apparent temperature is not commonly forecast or publicised as is actual temperature; hence, for health promotion workers and school staff to give advice to students in relation to temperatures, their source of temperature information being the media, actual temperature is likely more appropriate than apparent temperature. 

### 2.4. School and Classroom Descriptive Data

Principals were asked to complete a questionnaire that covered school infrastructure, uniform, scheduling and temperature-related factors. Specifically, they were asked to describe the topographical location of the school, typical school infrastructure and classroom building and roof type, school uniform and timetabling of physical education classes. In relation to temperature, principals were asked whether they had experienced a temperature-health related incident in the past year, whether they perceived that students were more productive during a particular season, and whether classrooms routinely had their windows left open, lights on and drinking water available in the room.

### 2.5. Student Questionnaire

The questionnaire (Figure S1) comprised two parts: demographics and possible confounders; and an hourly symptom log. Students first provided their grade, age and gender, the name of their school, whether they smoke, consume alcohol and/or exercise (factors known to affect personal susceptibility to hyperthermia) [25], and their weight and height. For each day, they then completed the hourly symptom log, agreeing (placing a tick or cross in the appropriate hourly slot) with as many symptoms that they were experiencing for each hour of the day between 8 h and 14 h (9 hourly period). The symptoms included tired, “hotter than normal”, “hot in the head”, “low concentration”, “sick”, “feeling dizzy”, “headache”, “nausea”, “fever”, “thirsty”, “sleepy”, “diarrhoea”, “hard to breathe” and “slow”.

### 2.6. Data Processing and Statistical Analysis

Temperature and relative humidity data were downloaded from the Lascar data loggers directly to computer via USB and saved in a .txt file. Principal questionnaire data were coded and entered electronically into Microsoft Excel. Student questionnaire data were also coded and entered electronically into Microsoft Excel. Student questionnaire data and temperature data were restructured into long format, cleaned and merged by date and time using Python (Python Software Foundation, Beaverton, OR, USA) before being imported into Stata 14.0 (StataCorp., College Station, TX, USA) [26] for analysis. Both principal and student questionnaire datasets underwent double data entry by trained data capturers. Unique identifier codes were used for schools and students.

School and student questionnaire data were analysed descriptively. Student differences in perceived heat-related health symptoms by non-modifiable and modifiable risk factors were explored using Random Effects (RE) panel data logistic regression models, all models controlled for school. The significance of these relationships were explored using *p*-values, odds ratios (OR), their corresponding 95% confidence intervals (CI) and the overall model Rho (ρ > 0.75 considered good). RE models were used so the time invariant variables (such as sex) remained in the models. Temperature and humidity data were plotted using time series analysis for each school by day of the week.

The relationships between temperature and heat-related health symptoms were explored on a bivariate level using RE panel data logistic regression models. The significance of these results were examined using *p*-values, ORs, their corresponding 95% CIs and the overall model Rho. These relationships were further explored controlling for other variables (time of day, sex, age, school, smoking, drinking and level of physical activity). Random effects models were used here too in order to retain the time invariant variables such as sex. The same models were also run for temperatures greater than or equal to 32 °C, the temperature above which personal metabolic rate at a given activity level is no longer stable [27] and discomfort and/or heat-health symptoms may occur (although they may occur at lower temperatures too, depending on personal susceptibility).

## 3. Results

### 3.1. Sample Description

Eight schools took part in the study and a total of 252 students completed the 5 day daily heat-health symptom log (Table 1). Of the total students in the class per school (Table 1, row 4) there was an 81.4% student response rate with 1260 student-days included in the analysis. Table 1 describes the variation in student gender, age and grade by school and overall. More girls (*n* = 159, 64.63%) than boys (*n* = 87, 35.37%) participated (6 students did not report their gender). The majority of students were in Grade 12 and aged 16.95 years.

More students agreed that they did not consume alcohol (*n* = 207, 84.15%) and smoke (*n* = 236, 94.4%) *vs.* those who did consume alcohol and did smoke, respectively. More students agreed that they exercised (*n* = 145, 63.3%) compared to those who said that they did not exercise. Although 48.41% and 65.87% of learners did not provide their weight and height, respectively, according to the responses received the average weight was 55.29 kg and average height was 1.54 metres.

### 3.2. Indoor Classroom Temperatures

**Figure 2:**
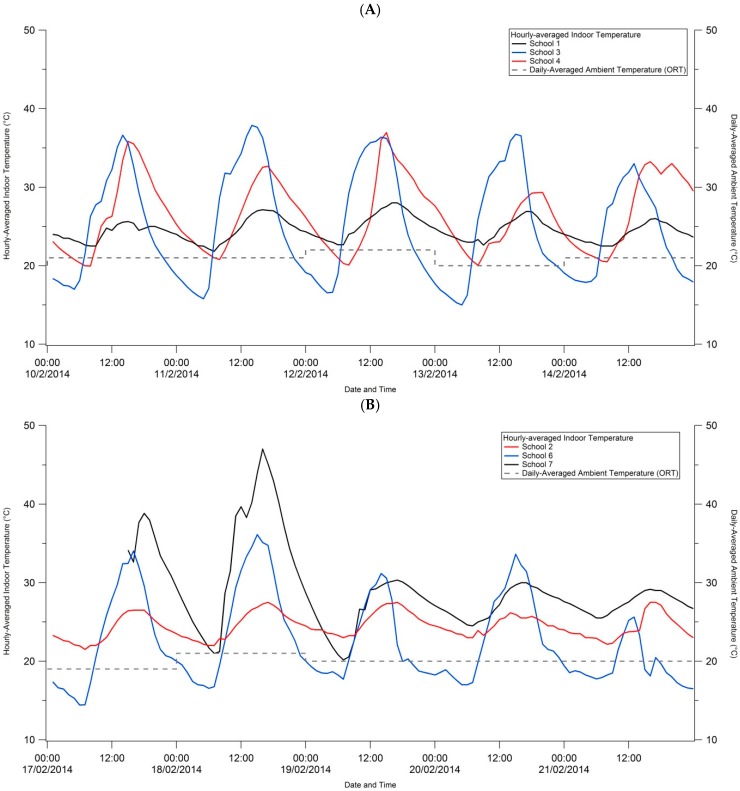

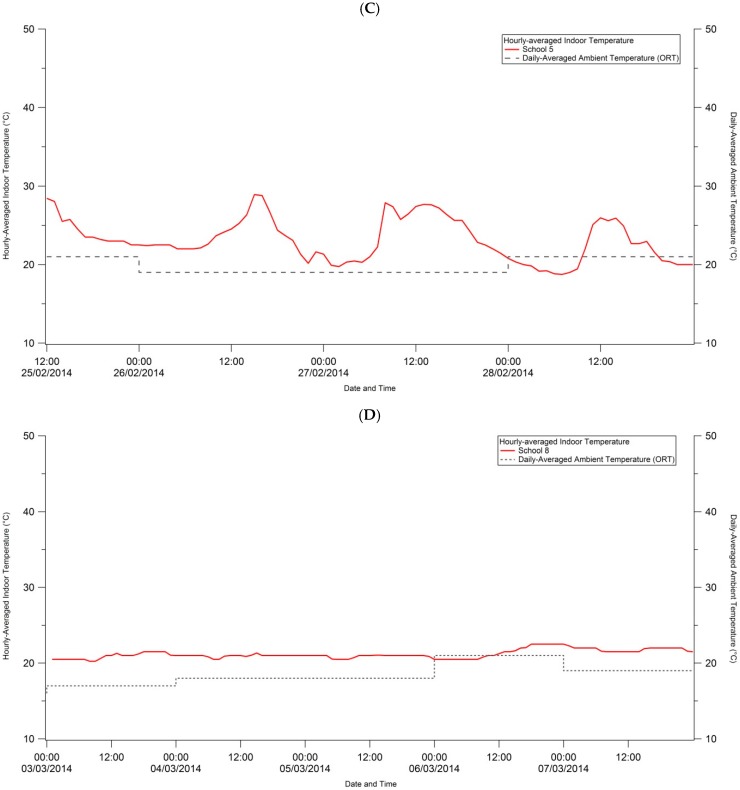
Indoor classroom temperatures (°C) by hour of day for Monday to Friday recorded by the Lascar data loggers at the eight schools where (**A**) Schools 1, 3, 4; (**B**) Schools 2, 6, 7; (**C**) School 5; and (**D**) School 8. Daily averages of ambient temperature recorded at Oliver Reginald Tambo (ORT) International Airport are displayed in gray dotted lines on Figure 2A–D.

Indoor classroom temperatures for the five study days are visually depicted by school in Figure 2 to illustrate temporal trends in classroom temperature. The school study took place at different times due to the enrolling of schools and obtaining of studies, hence we grouped schools in the graphs by date in order to compare ambient temperature data by date. Generally, indoor classroom temperatures were between ~21 °C and 34 °C for all schools and all study days, except for School 7 where temperatures exceeded 40 °C on days 1 and 2. 

Mean daily temperature and humidity (±1 standard deviation) measurements are provided in Table 2a by school and study day, as well as overall means for temperature and humidity. School 7 experienced two days (17 and 18 February 2014) when the mean temperature exceeded 30 °C. Five of the schools (Schools 3, 4, 5, 6 and 7) experienced temperatures in excess of 30 °C at least once during the five study days. A delayed start at School 8 meant that the study was carried out here in autumn when outdoor ambient temperatures were cooler and more consistent in minimum and maximum daily range, hence the lack of diurnal variation.

Mean indoor classroom temperatures ranged between night time minimum temperatures of ~15 °C and daytime maximum temperatures of ~35 °C (Table 2b). Day time maximum temperatures ranged between 21 °C (School 8) and 47 °C (School 7). At Schools 3 and 7, maximum day time temperatures tended to occur (although inconsistently) above 30 °C. Compared to mean and maximum ambient outdoor temperature measured at the international airport in Johannesburg (Table S4), mean classroom temperatures were all warmer than the mean outdoor ambient temperatures except for School 6 on day 5. The container classroom at School 7 showed the largest difference in indoor and ambient mean temperatures with warmer indoor mean temperatures of 15.2 °C and 11.8 °C on study days 1 and 2, respectively. The difference between the 99th percentile indoor classroom temperature and the reported maximum ambient temperature for each school study day is also shown in Table S4. Here, indoor maximum temperatures in brick classrooms (Schools 1, 2, 4, 5, 6 and 8) were either slightly lower than the ambient maximum temperature, or 2 to 3 °C warmer. The most striking results were for the prefabricated classroom (School 3, all days) and the container classroom (School 7, days 1 and 2). The former showed differences in maximum classroom and ambient temperatures of either 7.5 °C and 10.0 °C, and the latter, 12.0 °C and 19.5 °C for days 1 and 2 respectively.

The calculated apparent temperatures (mean ± 1 Standard Deviation and range, 1st and 99th percentiles) for each school and study day in Table S5 are comparable to the indoor temperatures measured, however, differences do exist. Between the 10 and 14 February and the 17 and 21 February, calculated indoor apparent temperatures were higher (sometimes up to 7 °C higher) than indoor classroom temperatures. During the study period 24–28 February and 3–7 March, calculated indoor apparent temperatures were similarly higher than the indoor classroom temperatures but much smaller differences (between 1 °C and 2.5 °C) were noted. The highest 99th percentile apparent temperature of 53.8 °C was calculated for School 7 (container classroom) on day 2.

### 3.3. School Environment and Principal Responses

Descriptive statistics of each school as reported by the school principal are given in Table 3. Schools were topographically located in either an urban or rural area, and in a valley or on a hill. All schools had electricity, piped water and sanitation. Six of the schools’ classrooms were made of brick (Schools 1, 2, 4, 5, 6 and 8). The School 3 classroom was made of prefabricated asbestos sheeting with a metal, corrugated iron roof. The School 7 classroom was a converted shipping container made of metal.

In terms of temperature-related questions, all principals reported that their schools only had natural ventilation in the classrooms. All principals reported that teachers left classroom windows open during class time, left lights on and teachers allowed drinking water to be available to students during class time. Physical education lessons, typically taking place outdoors, were scheduled either between 10 h and 12 h (Schools 1, 3, 4 and 7) or at any time of the day (Schools 2, 5, 6 and 8). Principals at Schools 1 and 6 reported that they had experienced a heat stress incidence by a student during the past year. Six of the eight principals agreed that students were most productive during the wintertime compared to summertime (as reported by principals at Schools 1 and 3, only). 

### 3.4. Student Perceived Heat-Related Symptoms

Majority (*n* = 248, 98.4%) of the sampled students agreed to having had at least one heat-health related symptom during at least one hour on any study day during the course of the study (Table 4). The most students in absolute numbers reporting heat-health symptoms were from Schools 1 and 4; Schools 1, 5, 7 and 8 had all their participating students reporting at least one heat-health related symptom during at least one hour on any study day.

Overall, the most prevalent heat-health related symptom reported by students was “tired“ where 97.2% (*n* = 245/252) agreed that they felt tired at least once during the study. This symptom was followed by “low concentration” (*n* = 244/252, 96.8%) and “sleepy” (*n* = 237/252, 94.0%) as the second and third most commonly reported symptoms overall. “Hotter than normal” (*n* = 229/252, 90.9%) and “thirsty” (*n* = 223/252, 88.5%) were also commonly reported symptoms. The least frequently reported symptoms among the sample were “diarrhoea” (40.9%) and “nausea” (54.8%). There were very little noticeable differences in the proportion of children reporting heat-related symptoms by school.

To determine the possible effect of non-modifiable and modifiable risk factors on perceived heat-health symptoms reported by the students the researchers considered symptoms experienced by gender and age, and alcohol consumption, regular smoking and regular exercise, respectively (Table 5). There were no statistically significant differences for either ‘any symptom’ or individual symptoms by risk factor likely as a result of the large proportion of children reporting the heat-health symptoms. In the instance of reported symptom “diarrhoea” and age, while the *p*-value was 0.057 (marginally statistically significant), the CI includes 1, rendering the result no longer significant.

### 3.5. Measured Indoor Classroom Temperatures in Relation to Students’ Perceived Heat-Related Health Symptoms

The results of the RE models exploring heat-related health symptoms and temperature, continuous and hourly-averaged temperature ≥32 °C, are given in Table 6a and Table 6b, respectively. Most of the model Rho (ρ) values were low (~0.50), meaning only around 50% of the variation seen in the outcome can be explained by the factors included in the model. We suspect that several other factors, beside temperature and including stress, sleep deprivation and pre-existing disease or illness, among others, may play a role in affecting many of the heat-health symptoms that we measured in this study. Rho values increased marginally in the multivariate models. For the majority of the symptoms, an increase in temperature was statistically significantly associated with the symptom, however, for the majority this appeared to weaken in the multivariate models (based on OR and 95% CI tending to be closer to 1). In the model for temperatures ≥32 °C (Table 6b) controlled for hour and school (both as categorical variables) statistically significant relationships were found between temperatures ≥32 °C and “tired” (*p* = 0.001, OR = 1.10, CI = 1.04 to 1.16) and “hard to breathe” (*p* > 0.001, OR = 1.29, CI = 1.16 to 1.44).

## 4. Discussion 

The overall aim of this study was to assess schoolchildren’s perceived heat-health symptoms in relation to indoor temperature during school hours in the classroom environment. To date, data on indoor classroom temperatures in South Africa is only available for one pilot study [28]. Here, we provided additional data on classroom temperatures in South Africa, the only other known study of its kind to do so in South Africa, and additionally describe self-reported heat-health symptoms in relation to temperature. These data are particularly important in light of climate change predictions for southern Africa and global warming leading to warmer temperatures and increased occurrence of heat waves. The 2015/2016 El Niño affecting southern Africa, for example, has contributed to warmer temperatures and widespread drought in many parts of South Africa, in particular [29]. High ambient temperatures influence indoor temperatures, particularly in indoor settings without internal temperature control such as air conditioning.

Two study objectives were defined. First, we aimed to quantify indoor classroom temperatures in a sample of Johannesburg government schools. The study took place during southern hemisphere summer months of February and March when ambient temperatures are typically high. Except for the school (School 8) observed in March, all schools had afternoon classroom temperatures in excess of 25 °C and with 18 school study days (out of a possible 40 school-days) with afternoon classroom temperatures 30 °C or higher. The highest classroom temperature was 47.5 °C and was measured in a classroom constructed out of a converted shipping container. While the container had a door and windows that were reported by the principal as usually being left open, classroom temperatures of greater 30 °C were recorded on all study days except day 5 (when the maximum temperature in the container classroom was 29.5 °C). Most people feel comfortable when indoor air temperatures are between 20 °C and 27 °C and relative humidity ranges between 35% and 60% [30]. Here, humidity levels at all schools on all days were typically within the acceptable range for human comfort (except School 6, day 5 and School 8, day 1); however, the classroom maximum temperatures were regularly higher than considered comfortable for humans. These elevated temperatures possibly explain why a majority of the student participants in the study reported heat-health symptoms at least once (but far more frequently than once in most cases) a day every day during the study period. The comparison of measured indoor temperature and calculated apparent temperatures suggests that students may in fact have been experiencing thermal discomfort from temperatures higher than the measured indoor temperatures. Given the predicted increase in ambient apparent temperatures for the CoJ and an increase in stretches of hot days [6] one may find an even greater proportion of students reporting heat-health symptoms in future studies.

High air temperatures have been associated with illness [31,32] and in extreme cases, mortality [33,34]. In this study, a high proportion of children reported feeling tired and found it difficult to breathe when temperatures exceeded 32 °C. This was probably because of high class student numbers and low ventilation in the classrooms. Despite the principal reporting that windows and doors were left open to allow for air flow into and out of the classrooms, researcher observations during study visits to download temperature data were that teachers kept doors and windows closed because of noise, or because they no longer could be opened. Moreover, the size of the part of the window that could open was small relative to the size of the classroom. Several studies have found that warm environments are associated with feeling sick, tiredness/lethargy and headaches [35]. Concentration is also affected by warm temperatures [36,37] as we found with 96% of students reporting low concentration relatively consistently during the study days.

Despite there being three different types of school classrooms in the study, it was not possible to detect statistically significant differences in reported health symptoms by school as a result of the small number of schools in the different categories (*i.e.*, five brick, one asbestos sheeting, one container). It was also not possible to detect statistically significant differences in reported heat-health symptoms by gender and age, or students who reportedly consumed alcohol, smoked or exercised *vs.* those who did not. Majority of students reportedly did not smoke (93.6%) or drink alcohol (82.0%), while about half of the sample exercised regularly (56.8%). The former may be an underestimate of smoking and drinking prevalence as a result of the perceived assumption that these activities should not be undertaken by minors.

When considering student perceived heat symptoms for classroom temperatures greater than or equal to 32 °C, only two differences were detectable. This may have been because students were in fact feeling heat effects at temperatures lower than 32 °C. However, there was likely a Hawthorne effect on symptom log completion, and other inaccuracies as a result of completing it under instruction by the teacher, or unreliably completing the log after the hour of the day has occurred introducing recall bias.

This study and its results should be substantiated with further longitudinal data. The most insightful learnings from this research were noting important steps that should be made when assessing perceived heat-health symptoms among students. Here, we found that self-report and completion of symptom logs over the period of one week is long and may have reduced compliance. A shorter period of self-report may be preferable, or the use of mobile phones or tablets to capture these data may encourage students to complete the logs without reporting fatigue. No verification of symptoms and physiological effects were made. Also, other factors may have contributed towards the health symptoms experienced by the children, for example illness, sleep deprivation and stress. It is not possible for our results to prove causation between temperature and heat-related symptoms. Future studies should consider including a health status questionnaire for students’ parents/caregivers to complete to obtain information on pre-existing disease, stress and current medication, for example. It would have been helpful if we had measured the body temperatures of the children each day during the study, as well as the outdoor ambient temperatures at the school. The principal questionnaire data were not sufficiently detailed to analyse for school infrastructural differences in relation to student perceived heat-health symptoms. A researcher-based observation sheet to capture details on classroom building materials, window size and ventilation use while class is in session would be preferable. In our study, the latter may be constrained by the Hawthorne effect since principals may have responded that windows and doors were kept open during class when in fact they were closed. Since each school operates according to their own set of rules and teacher preferences, a larger study to gather information on ventilation may be important. There were fewer Grade 8 and 9 students included in the study compared to students from Grades 10, 11 and 12. A delayed start at School 8 meant that the study was carried out here in autumn and not summer, and additionally, the class in which the study was carried out only had 8 students. However, we included these data for comparison purposes.

## 5. Conclusions

Heat-related health impacts and perceived health symptoms are potentially a problem especially in public schools with limited resources. Here, we found that a high proportion of students felt tired, had low concentration and felt sleepy during at least one hour on any day during summer. Some recommendations may be made based on these panel study findings. School classrooms made of prefabricated asbestos sheeting with metal roofs or converted shipping containers are more likely to experience high indoor temperatures compared to brick classroom when occupied by a similar number of students. Whenever possible, classrooms made from non-brick materials, such as asbestos and metal, should be temporary with the goal towards a brick classroom preferred and supported by the authorities. No information at school, city or national level describe comprehensively the building type and related infrastructural details of school buildings or classroom ventilation strategies; such a database would be extremely useful to better understand heat health risks that schoolchildren may face.

Ventilation in classrooms should be a priority and the installation of ceiling fans, so long as electricity supply is reliable, may assist in cooling classrooms with open windows and doors during warm weather. Water should continue to be available to students during classroom hours to ensure they remain hydrated especially when classroom temperatures are high. A more comprehensive, longitudinal study with a larger sample of students and schools, and with validation methods in the study design is necessary to confirm the findings observed here and provide the necessary evidence for urgent policy development on heat, health and schools in South Africa and southern Africa.

## Figures and Tables

**Table 1:** Sample descriptive statistics by school and overall for the complete sample.

Variable	School 1	School 2	School 3	School 4	School 5	School 6	School 7	School 8	All (n)	All (%)
Region	F	F	F	B	B	G	G	G		
School roll size (n)	800	1235	472	1500	888	1900	1874	1807	10,476	100.0
Class roll size (n)	49	38	29	50	33	45	35	30	307	-
Total per class in study (n)	47	38	26	45	29	30	29	8	252	81.4
% of class in study	95.9	100.0	89.6	90.0	87.8	66.6	82.8	26.6	-	79.9(AVERAGE)
School study dates (2014)	10–14 Feb.	17–21 Feb.	10–14 Feb.	10–14 Feb.	24–28 Feb.	17–21 Feb.	17–21 Feb.	3–7 Mar.	-	-
Gender										
Male (n)	16	18	3	18	12	11	7	2	86	34.4
Female (n)	31	20	21	26	17	16	22	6	158	63.2
*Missing (n)*	*0*	*0*	*2*	*1*	*0*	*3*	*0*	*0*	*6*	*2.4*
Age										
14 years (n)	1	1	0	3	0	1	0	0	5	2.02
15 years (n)	9	3	3	9	2	4	0	0	30	12.1
16 years (n)	8	4	9	21	3	13	0	0	58	23.4
17 years (n)	11	14	11	8	13	6	9	1	73	29.5
18 years (n)	10	12	1	2	5	5	11	4	50	20.2
>18 years (n)	8	4	0	0	6	1	9	3	31	12.5
*Missing (n)*	*0*	*0*	*2*	*2*	*0*	*0*	*0*	*0*	*4*	*1.6*
Grade										
Grade 8 (n)	0	1	0	0	0	0	0	0	1	0.4
Grade 9 (n)	0	1	2	1	0	0	0	0	4	1.5
Grade 10 (n)	25	0	0	32	0	8	0	0	65	25.7
Grade 11 (n)	0	13	24	12	14	21	0	0	84	33.3
Grade 12 (n)	22	23	0	0	15	1	29	8	98	38.8
*Missing (n)*	*0*	*0*	*0*	*0*	*0*	*0*	*0*	*0*	*0*	*0*
Do you consume alcohol?										
No (n)	38	26	19	34	28	28	26	8	205	82.0
Yes (n)	9	11	4	11	1	1	2	0	39	15.6
*Missing (n)*	*0*	*1*	*3*	*0*	*0*	*1*	*1*	*0*	*6*	*2.4*
Do you smoke?										
No (n)	42	34	24	44	28	27	29	8	234	93.6
Yes (n)	5	4	2	1	1	3	0	0	16	6.4
*Missing (n)*	*0*	*0*	*0*	*0*	*0*	*0*	*0*	*0*	*0*	*0*
Do you exercise?										
No (n)	1	8	9	16	13	15	18	5	85	41.2
Yes (n)	31	28	15	28	15	14	10	3	142	56.8
*Missing (n)*	*15*	*2*	*2*	*1*	*1*	*1*	*1*	*0*	*23*	*2.0*

**Table 2a:** Mean daily indoor temperature concentrations and mean daily indoor relative humidity levels (±1 Standard Deviation, SD) by school and study day.

School No. (Dates)	Day 1		Day 2		Day 3		Day 4		Day 5		Overall	
	Mean		Mean		Mean		Mean		Mean		Mean	
	Temperature °C ± 1 SD	Humidity% ± 1 SD	Temperature °C ± 1 SD	Humidity% ± 1 SD	Temperature °C ± 1 SD	Humidity% ± 1 SD	Temperature °C ± 1 SD	Humidity% ± 1 SD	Temperature °C ± 1 SD	Humidity% ± 1 SD	Temperature °C	Humidity%
1 (10–14 Feb.)	24.1 ± 0.9	55.9 ± 2.7	24.5 ± 1.7	41.6 ± 9.3	25.3 ± 1.7	34.2 ± 4.6	24.5 ± 1.2	47.4 ± 6.6	24.0 ± 1.1	49.8 ± 9.5	24.5	45.8
2 (17–21 Feb.)	23.9 ± 2.6	37.7 ± 6.6	24.5 ± 1.8	41.2 ± 5.0	25.0 ± 1.4	49.3 ± 3.1	24.4 ± 0.9	53.5 ± 2.4	24.1 ± 1.6	55.3 ± 4.5	24.4	47.4
3 (10–14 Feb.)	24.8 ± 6.4	56.5 ± 16.9	26.1 ± 7.6	43.7 ± 17.6	25.8 ± 7.4	37.5 ± 12.7	24.7 ± 7.4	55.9 ± 17.9	23.8 ± 5.4	54.8 ± 18.7	25.0	49.7
4 (10–14 Feb.)	26.6 ± 5.1	52.4 ± 3.7	26.4 ± 3.8	46.8 ± 2.0	27.2 ± 5.2	42.4 ± 2.7	24.9 ± 2.8	38.7 ± 1.2	26.9 ± 4.9	40.9 ± 1.9	26.4	44.2
5 (24–28 Feb.)	**^#^**	**^#^**	24.2 ± 1.9	44.4 ± 5.4	23.5 ± 2.2	53.7 ± 5.3	23.8 ± 2.9	57.3 ± 7.8	21.4 ± 2.4	67.7 ± 7.0	23.1	57.3
6 (17–21 Feb.)	22.8 ± 6.3	47.1 ± 15.4	24.8 ± 6.7	46.5 ± 12.8	22.0 ± 4.5	63.1 ± 12.5	23.5 ± 5.4	59.0 ± 15.7	19.3 ± 2.6	77.6 ± 7.9	22.5	58.7
7 (17–21 Feb.)	34.2 ± 3.1	36.2 ± 3.5	32.8 ± 8.1	31.6 ± 8.6	26.5 ± 3.3	32.0 ± 5.9	27.3 ± 1.8	39.0 ± 3.8	27.3 ± 1.1	40.6 ± 4.9	29.0	35.8
8 (3–7 Mar.)	20.9 ± 0.4	70.7 ± 3.5	20.9 ± 0.1	74.5 ± 2.1	20.8 ± 0.2	78.1 ± 2.9	21.4 ± 0.8	74.3 ± 3.9	21.8 ± 0.2	70.4 ± 0.6	21.1	73.6

**^#^** Missing data. No data were recorded on day 1 for school 5; recordings only began on day 2.

**Table 2b:** Range in indoor classroom temperature concentrations (1st and 99th percentiles) by school and study day.

School No.	Study Day 1		Study Day 2		Study Day 3		Study Day 4		Study Day 5		Overall—All Study Days	
	Temperature °C		Temperature °C		Temperature °C		Temperature °C		Temperature °C		Temperature °C	
	1st	99th	1st	99th	1st	99th	1st	99th	1st	99th	1st	99th
1	22.5	26.5	21.5	27.5	22.5	28.0	22.5	27.0	22.0	26.0	21.5	28.0
2	21.5	26.5	22.0	27.5	23.0	27.5	23.0	26.5	22.0	27.5	21.5	27.5
3	17.0	37.0	15.5	38.0	16.5	36.5	15.0	37.0	17.5	33.5	15.0	38.0
4	19.5	36.5	20.5	33.0	20.0	37.5	20.0	29.5	20.5	33.5	19.5	37.5
5	**^#^**	**^#^**	22.5	30.5	20.0	29.5	19.5	29.5	18.5	27.0	18.5	31.5
6	14.0	34.5	15.5	37.0	17.5	32.5	17.0	34.5	16.5	28.5	14.0	37.0
7	28.5	39.0	21.0	47.5	20.0	31.5	24.5	30.0	25.5	29.5	20.0	47.5
8	20.0	21.5	20.5	21.5	20.5	21.5	20.5	22.5	21.5	22.5	20.0	22.5

**^#^** Missing data. No data were recorded on day 1 for school 5; recordings only began on day 2.

**Table 3:** Description of each school’s infrastructure and principal heat-related questions.

Variables	School 1	School 2	School 3	School 4	School 5	School 6	School 7	School 8
**School location and infrastructure**								
Topographical location of school	Urban in a valley	Urban area	Surrounded by trees, plants, urban area	Industrial area, surrounded by trees and plants urban area	Urban area	Hilltop rural area	In a valley rural area	Hilltop urban area
Piped water	Yes	Yes	Yes	Yes	Yes	Yes	Yes	Yes
Sanitation	Yes	Yes	Yes	Yes	Yes	Yes	Yes	Yes
Electricity	Yes	Yes	Yes	Yes	Yes	Yes	Yes	Yes
Building material of classrooms	Brick	Brick	Prefabricated walls from asbestos sheeting and metal/corrugated iron roof	Brick	Brick	Brick	Container make of metal	Brick
**Temperature-related questions**								
Type of uniforms	Tracksuits, skirts, trousers with shirt	Blazer pants shirts tie	Winter: blazer shirts trousers Summer: trousers shirts	Trousers skirt Shirt pull over	Trousers shirts	Tracksuits skirts trousers shirt	(Missing)	Jerseys blazers shirts dungaree trousers
Physical education time	10:00–12:00	Anytime	10:00–12:00	10:00–12:00	Anytime	Anytime	10:00–12:00	Anytime
Ventilation	Natural	Natural	Natural	Natural	Natural	Natural	Natural	Natural
	Windows open	Windows open	Windows open	Windows open	Windows open	Windows open	Windows open	Windows open
Lighting	Lights on	Lights on	Lights on	Lights on	Lights on	Lights on	Lights on	Lights on
Teachers allowed drinking water to be available to students during class time	Yes	Yes	Yes	Yes	Yes	Yes	Yes	Yes
Heat stress incidence in past year	Yes	No	No	No	No	Yes	No	No
Perception of learners most productive season	Summer	Winter	Summer	Winter	Winter	Winter	Winter	Winter

**Table 4:** Perceived heat-health symptoms experienced by the students by school and by symptom (any symptom and individual symptoms) at any point during the time period.

Symptom	School 1 n (%)	School 2 n (%)	School 3 n (%)	School 4 n (%)	School 5 n (%)	School 6 n (%)	School 7 n (%)	School 8 n (%)	All Students n (%)
Total students (n)	47	38	26	45	29	30	29	8	252
All symptoms combined	47 (100.0)	36 (94.7)	25 (96.2)	44 (97.8)	29 (100.0)	30 (100.0)	29 (100)	8 (100.0)	248 (98.4)
Individual symptoms									
Tired	47 (100.0)	36 (100.0)	25 (42.3)	43 (95.6)	29 (100.0)	29 (66.7)	28 (96.6)	8 (100.0)	245 (97.2)
Hotter than normal	47 (100.0)	35 (92.2)	25 (38.4)	42 (93.3)	24 (82.8)	25 (83.3)	24 (82.8)	7 (87.5)	229 (90.9)
Hot in the head	43 (91.5)	31 (86.1)	21 (30.7)	36 (80.0)	24 (82.8)	27 (90.0)	25 (86.2)	5 (62.5)	212 (84.1)
Low concentration	47 (100.0)	36 (100.0)	25 (38.4)	42 (93.3)	29 (100.0)	28 (93.3)	29 (100.0)	8 (100.0)	244 (96.8)
Sick	43 (91.5)	29 (80.6)	19 (19.2)	35 (77.8)	24 (82.8)	24 (80.0)	26 (89.7)	6 (75.0)	206 (81.7)
Feeling dizzy	38 (80.9)	29 (80.6)	20 (11.5)	33 (73.3)	25 (86.2)	24 (80.0)	20 (69.0)	3 (37.5)	192 (76.2)
Headache	41 (87.2)	33 (91.7)	20 (30.7)	37 (82.2)	23 (79.3)	25 (83.3)	27 (93.1)	7 (87.5)	213 (84.5)
Nausea	26 (55.3)	23 (63.9)	16 (3.8)	22 (48.9)	19 (65.5)	16 (53.3)	14 (48.3)	2 (25)	138 (54.8)
Fever	34 (91.5)	25 (69.5)	16 (11.5)	27 (60.0)	16 (55.2)	21 (70.0)	20 (69.0)	5 (62.5)	164 (65.1)
Thirsty	46 (97.9)	35 (92.2)	23 (30.7)	40 (88.9)	25 (86.2)	21 (70.0)	26 (89.7)	7 (87.5)	223 (88.5)
Sleepy	46 (97.9)	36 (100.0)	24 (38.4)	39 (86.7)	29 (100.0)	27 (90.0)	28 (96.6)	8 (100.0)	237 (94.1)
Diarrhoea	20 (42.6)	15 (41.7)	7 (7.6)	14 (31.1)	17 (58.6)	11 (36.7)	14 (48.3)	5 (62.5)	103 (40.9)
Hard to breathe	28 (59.6)	21 (58.3)	18 (7.6)	25 (55.6)	19 (65.5)	16 (53.3)	14 (48.3)	4 (50.0)	145 (57.5)
Slow	44 (93.6)	31 (86.1)	24 (30.7)	36 (80.0)	27 (93.1)	24 (80.0)	28 (96.6)	8 (100.0)	222 (88.1)

**Table 5:** Differences in perceived heat-related health symptoms of students by non-modifiable and modifiable risk factors (controlled for school).

Symptom	Non-Modifiable Risk Factor						Modifiable Risk Factor								
	Gender ^a,#^			Age ^b^			Regularly Consumes Alcohol ^c^			Regularly Smokes ^d^			Regularly Exercises ^e^		
	*p*-value	OR (95% CI)	ρ	*p*-value	OR (95% CI)	ρ	*p*-value	OR (95% CI)	ρ	*p*-value	OR (95% CI)	ρ	*p*-value	OR (95% CI)	ρ
Any symptom	0.511	0.86 (0.55–1.34)	0.44	0.721	0.97 (0.83–1.14)	0.45	0.597	0.86 (0.50–1.49)	0.44	0.710	0.86 (0.39–1.89)	0.45	0.510	1.18 (0.72–1.92)	0.48
Individual symptoms															
Tired	0.804	0.97 (0.73–1.28)	0.23	0.475	1.01 (0.94–1.15)	0.23	0.970	1.01 (0.80–1.45)	0.23	0.220	0.71 (0.41–1.23)	0.23	0.037	0.72 (0.53–0.98)	0.24
Hotter than normal	0.787	0.95 (0.67–1.35)	0.32	0.445	0.95 (0.84–1.08)	0.32	0.586	0.88 (0.55–1.40)	0.32	0.827	0.93 (0.47–1.83)	0.31	0.749	0.94 (0.64–1.38)	0.33
Hot in the head	0.440	1.17 (0.78–1.76)	0.38	0.364	0.94 (0.81–1.08)	0.38	0.461	0.81 (0.47–1.40)	0.38	0.492	1.33 (0.59–2.96)	0.38	0.743	1.08 (0.69–1.70)	0.39
Low concentration	0.164	0.80 (0.58–1.10)	0.29	0.425	1.05 (0.94–1.17)	0.28	0.778	0.94 (0.62–1.43)	0.29	0.371	0.75 (0.39–1.42)	0.29	0.125	0.75 (0.53–1.08)	0.30
Sick	0.948	0.98 (0.63–1.55)	0.43	0.596	0.96 (0.82–1.12)	0.43	0.710	0.89 (0.48–1.64)	0.44	0.332	0.63 (0.24–1.61)	0.43	0.650	0.89 (0.53–1.49)	0.46
Feeling dizzy	0.463	1.18 (0.76–1.85)	0.42	0.157	0.89 (0.76–1.04)	0.42	0.705	1.12 (0.62–2.04)	0.43	0.633	0.80 (0.32–2.01)	0.42	0.472	1.20 (0.73–2.00)	0.45
Headache	0.936	1.02 (0.67–1.55)	0.40	0.178	1.12 (0.95–1.29)	0.40	0.420	1.26 (0.72–2.20)	0.41	0.293	0.63 (0.26–1.50)	0.41	0.669	1.11 (0.69–1.80)	0.43
Nausea	0.882	1.05 (0.57–1.94)	0.56	0.636	0.95 (0.76–1.19)	0.57	0.690	0.84 (0.35–1.99)	0.58	0.476	1.60 (0.44–5.78)	0.58	0.551	1.25 (0.60–2.58)	0.60
Fever	0.651	0.87 (0.48–1.58)	0.56	0.639	0.95 (0.77–1.18)	0.57	0.891	0.94 (0.42–2.14)	0.58	0.202	2.19 (0.66–7.32)	0.57	0.159	1.66 (0.82–3.37)	0.60
Thirsty	0.776	1.06 (0.70–1.60)	0.40	0.943	0.99 (0.86–1.15)	0.40	0.926	1.02 (0.59–1.78)	0.40	0.751	0.88 (0.39–1.99)	0.40	0.927	0.99 (0.62–1.59)	0.43
Sleepy	0.508	1.12 (0.80–1.56)	0.31	0.274	1.07 (0.95–1.21)	0.31	0.907	1.03 (0.66–1.60)	0.31	0.232	0.67 (0.34–1.29)	0.31	0.148	0.75 (0.51–1.11)	0.33
Diarrhoea	0.831	1.09 (0.49–2.46)	0.69	0.057	1.32 (0.99–1.76)	0.69	0.490	1.46 (0.50–4.23)	0.69	0.332	2.15 (0.46–10.15)	0.69	1.000	1.00 (0.40–2.49)	0.71
Hard to breathe	0.951	0.98 (0.53–1.82)	0.56	0.194	0.86 (0.63–1.08)	0.57	0.606	1.24 (0.55–2.82)	0.57	0.251	2.02 (0.61–6.68)	0.57	0.031	2.19 (1.08–4.44)	0.58
Slow	0.720	0.93 (0.63–1.37)	0.36	0.622	1.03 (0.90–1.19)	0.36	0.751	0.92 (0.55–1.54)	0.36	0.817	0.91 (0.43–1.95)	0.36	0.432	0.84 (0.55–1.29)	0.34

**^#^** Reference groups for ^a^ is male; **^b^** age is continuous so no reference group; **^c^** is “no alcohol consumption”; **^d^** is “does not smoke regularly”; and **^e^** is “does not exercise regularly”.

**Table 6a:** Results of panel data logistic regression models exploring the number of students agreeing with a symptom *versus* temperature for the matching interval when symptom was reported, for two models uncontrolled and controlled *.

Symptom	Uncontrolled			Controlled		
	*p*-value	OR (95% CI) ^#^	ρ	*p*-value	OR (95% CI)	ρ
All symptoms	<0.001	1.11 (1.09–1.12)	0.48	<0.001	1.10 (1.08–1.12)	0.50
Individual symptoms						
Tired	<0.001	1.09 (1.09–1.11)	0.24	<0.001	1.06 (1.04–1.08)	0.25
Hotter than normal	<0.001	1.13 (1.11–1.14)	0.31	<0.001	1.08 (1.06–1.10)	0.34
Hot in the head	<0.001	1.10 (1.08–1.11)	0.38	<0.001	1.07 (1.05–1.10)	0.40
Low concentration	<0.001	1.07 (1.06–1.08)	0.28	<0.001	1.05 (1.03–1.07)	0.30
Sick	0.077	1.01 (1.00–1.03)	0.45	0.485	1.01 (0.99–1.03)	0.47
Feeling dizzy	<0.001	1.05 (1.03–1.06)	0.42	0.102	1.02 (1.00–1.04)	0.45
Headache	<0.001	1.06 (1.04–1.07)	0.42	<0.001	1.03 (1.01–1.05)	0.44
Nausea	0.367	1.01 (0.99–1.03)	0.56	0.093	1.03 (1.00–1.06)	0.57
Fever	<0.001	1.04 (1.03–1.06)	0.58	<0.001	1.05 (1.03–1.07)	0.58
Thirsty	<0.001	1.06 (1.05–1.08)	0.41	0.001	1.03 (1.01–1.05)	0.43
Sleepy	<0.001	1.06 (1.05–1.07)	0.32	<0.001	1.03 (1.02–1.05)	0.33
Diarrhoea	0.005	1.03 (1.01–1.06)	0.70	0.359	1.02 (0.98–1.05)	0.72
Hard to breathe	<0.001	1.06 (1.04–1.08)	0.57	<0.001	1.07 (1.04–1.10)	0.57
Slow	<0.001	1.07 (1.06–1.09)	0.37	0.001	1.03 (1.01–1.05)	0.39

* Controlling for time of day, sex, age, school, smoking, drinking and level of physical activity; **^#^** Temperature variable is continuous so no reference group.

**Table 6b:** Results of panel data logistic regression models exploring the number of students agreeing with a symptom *vs.* temperature ≥ 32 °C for the matching interval when symptom was reported, for two models uncontrolled and controlled *.

Symptom	Uncontrolled			Controlled		
	*p*-value	OR (95% CI) ^#^	ρ	*p*-value	OR (95% CI)	ρ
All symptoms	0.821	1.01 (0.94–1.09)	0.68	0.319	1.04 (0.96–1.13)	0.68
Individual symptoms						
Tired	0.001	1.10 (1.04–1.15)	0.46	0.001	1.10 (1.04–1.16)	0.43
Hotter than normal	0.783	1.01 (0.95–1.06)	0.49	0.657	1.01 (0.95–1.08)	0.46
Hot in the head	0.262	1.03 (0.98–1.09)	0.51	0.137	1.05 (0.99–1.12)	0.49
Low concentration	0.680	0.99 (0.94–1.04)	0.43	0.228	1.04 (0.98–1.11)	0.41
Sick	0.004	1.10 (1.03–1.18)	0.65	0.044	1.08 (1.00–1.17)	0.64
Feeling dizzy	0.460	1.03 (0.96–1.10)	0.53	0.402	1.03 (0.96–1.12)	0.52
Headache	0.086	1.05 (0.99–1.11)	0.52	0.212	1.04 (0.98–1.11)	0.51
Nausea	0.110	1.07 (0.98–1.18)	0.70	0.114	1.09 (0.98–1.22)	0.69
Fever	0.517	1.02 (0.95–1.10)	0.70	0.986	1.00 (0.92–1.08)	0.70
Thirsty	0.166	0.96 (0.91–1.02)	0.54	0.125	0.95 (0.90–1.01)	0.53
Sleepy	0.121	0.96 (0.90–1.01)	0.59	0.569	0.98 (0.92–1.05)	0.58
Diarrhoea	0.085	1.09 (0.99–1.20)	0.80	0.154	1.09 (0.97–1.21)	0.81
Hard to breathe	<0.001	1.19 (1.09–1.29)	0.68	<0.001	1.29 (1.16–1.44)	0.67
Slow	0.332	1.03 (0.97–1.08)	0.48	0.151	1.05 (0.98–1.11)	0.47

* Controlling for time of day, sex, age, school, smoking, drinking and level of physical activity; **^#^** Temperature variable is continuous so no reference group.

## Supplementary Materials

The following are available online at www.mdpi.com/1660-4601/13/6/566/s1. Figure S1: Student questionnaire; Table S1: Mean daily ambient temperature and humidity data recorded at the OR Tambo International Airport weather station in the City of Johannesburg for the study days; Table S2: Minimum and maximum daily ambient temperature data recorded at the OR Tambo International Airport weather station in the City of Johannesburg for the study days; Table S3: Minimum and maximum daily ambient humidity data recorded at the OR Tambo International Airport weather station in the City of Johannesburg for the study days; Table S4: Difference between indoor classroom temperatures (mean and 99th percentile) and ambient outdoor temperatures measured at the OR Tambo International Airport weather station; Table S5: Mean (±1 Standard Deviation, SD) and range (1st and 99th percentile) of daily indoor apparent temperature calculated using measured indoor temperature and relative humidity readings at the eight schools by school and study day.
