# Supplementary material for: Students’ Perceived Heat-Health Symptoms Increased with Warmer Classroom Temperatures
Source: Int J Environ Res Public Health. 2016 Jun 7;13(6):566. doi: 10.3390/ijerph13060566 (PMC4924023; doi:10.3390/ijerph13060566)
Supplement: Supplementary file 1 [file ijerph-13-00566-s001.pdf]

Supplementary Materials: Students' Perceived Heat-Health Symptoms Increased with Warmer Classroom Temperatures

Shalin Bidassey-Manilal, Caradee Y. Wright, Jacobus C. Engelbrecht, Patricia N. Albers, Rebecca M. Garland and Mamopeli Matooane

Table S1. Mean daily ambient temperature and humidity data recorded at the OR Tambo International Airport weather station in the City of Johannesburg for the study days.

School No.	Dates	Study Day 1		Study Day 2		Study Day 3		Study Day 4		Study Day 5	
		Temperature °C	Humidity %	Temperature °C	Humidity %	Temperature °C	Humidity %	Temperature °C	Humidity %	Temperature °C	Humidity %
1	10–14 Feb.	21 *	64	21	50	22	40	20	64	21	59
2	17–21 Feb.	19	50	21	49	20	63	20	64	20	83
3	10–14 Feb.	21	64	21	50	22	40	20	64	21	59
4	10–14 Feb.	21	64	21	50	22	40	20	64	21	59
5	24–28 Feb.	20	73	21	70	19	77	19	68	21	55
6	17–21 Feb.	19	50	21	49	20	63	20	64	20	83
7	17–21 Feb.	19	50	21	49	20	63	20	64	20	83
8	3–7 Mar.	17	90	18	89	18	89	21	76	19	82

Note. * Data were not provided with decimal points.

Table S2. Minimum and maximum daily ambient temperature data recorded at the OR Tambo International Airport weather station in the City of Johannesburg for the study days.

School No.	Dates	Study Day 1		Study Day 2		Study Day 3		Study Day 4		Study Day 5	
		Temperature °C		Temperature °C		Temperature °C		Temperature °C		Temperature °C	
		Min.	Max.	Min.	Max.	Min.	Max.	Min.	Max.	Min.	Max.
1	10–14 Feb.	16 *	27	14	28	16	29	12	27	16	26
2	17–21 Feb.	11	27	14	28	14	26	14	26	15	25
3	10–14 Feb.	16	27	14	28	16	29	12	27	16	26
4	10–14 Feb.	16	27	14	28	16	29	12	27	16	26
5	24–28 Feb.	15	24	15	26	15	23	14	24	16	25
6	17–21 Feb.	11	27	14	28	14	26	14	26	15	25
7	17–21 Feb.	11	27	14	28	14	26	14	26	15	25
8	3–7 Mar.	15	18	16	19	16	19	17	24	16	23

Note. * Data were not provided with decimal points.

Table S3. Minimum and maximum daily ambient humidity data recorded at the OR Tambo International Airport weather station in the City of Johannesburg for the study days.

School No.	Dates	Study Day 1 Humidity (%)		Study Day 2 Humidity (%)		Study Day 3 Humidity (%)		Study Day 4 Humidity (%)		Study day 5 Humidity (%)	
		Min.	Max.	Min.	Max.	Min.	Max.	Min.	Max.	Min.	Max.
1	10–14 Feb.	37 *	88	23	88	19	68	36	94	28	88
2	17–21 Feb.	26	82	21	77	37	94	39	88	60	94
3	10–14 Feb.	37	88	23	88	19	68	36	94	28	88
4	10–14 Feb.	37	88	23	88	19	68	36	94	28	88
5	24–28 Feb.	50	88	33	94	49	94	40	94	39	73
6	17–21 Feb.	26	82	21	77	37	94	39	88	60	94
7	17–21 Feb.	26	82	21	77	37	94	39	88	60	94
8	3–7 Mar.	82	94	78	94	80	94	52	94	55	94

Note. * Data were not provided with decimal points.

Table S4. Difference between indoor classroom temperatures (mean and 99th percentile) and ambient outdoor temperatures measured at the OR Tambo International Airport weather station.

School No.	Dates	Study Day 1		Study Day 2		Study Day 3		Study Day 4		Study Day 5	
		Difference in Mean	Difference in Max.	Difference in Mean	Difference in Max.	Difference in Mean	Difference in Max.	Difference in Mean	Difference in Max.	Difference in Mean	Difference in Max.
1	10–14 Feb.	3.1	-0.5	3.5	−0.5	3.3	−1.0	4.5	0.0	3.0	0.0
2	17–21 Feb.	4.9	-0.5	3.5	−0.5	5.0	1.5	4.4	0.5	4.1	2.5
3	10–14 Feb.	3.8	10.0	5.1	10.0	3.8	7.5	4.7	10.0	2.8	7.5
4	10–14 Feb.	5.6	9.5	5.4	5.0	5.2	8.5	4.9	2.5	5.9	7.5
5	24–28 Feb.	#	#	3.2	4.5	4.5	6.5	4.8	5.5	0.4	2.0
6	17–21 Feb.	3.8	7.5	3.8	9.0	2.0	6.5	3.5	8.5	−0.7	3.6
7	17–21 Feb.	15.2	12.0	11.8	19.5	6.5	5.5	7.3	4.0	7.3	4.5
8	3–7 Mar.	3.9	3.5	2.9	2.5	2.8	2.5	0.4	−1.5	2.8	−0.5

Note. [#] Missing data. No data were recorded on day 1 for School 5; recordings only began on day 2.

Table S5. Mean (±1 Standard Deviation, SD) and range (1st and 99th percentile) of daily indoor apparent temperature calculated using measured indoor temperature and relative humidity readings at the eight schools by school and study day.

School No. (dates)	Day 1			Day 2			Day 3			Day 4			Day 5		
	Temperature °C ± 1 SD	1st	99th	Temperature °C ± 1 SD	1st	99th	Temperature °C ± 1 SD	1st	99th	Temperature °C ± 1 SD	1st	99th	Temperature °C ± 1 SD	1st	99th
1 (10–14 Feb.)	25.7 ± 1.1	23.7	28.1	24.7 ± 1.3	21.9	27.1	24.9 ± 1.6	22.3	27.8	25.3 ± 1.4	23.2	27.6	25.8 ± 0.6	23.5	26.2
2 (17–21 Feb.)	23.5 ± 1.4	21.4	25.8	24.7 ± 1.8	21.9	27.7	26.2 ± 1.6	23.6	29.0	25.9 ± 1.0	24.2	28.4	25.6 ± 1.9	23.3	29.8
3 (10–14 Feb.)	26.4 ± 7.1	18.2	39.6	26.5 ± 7.9	15.7	38.7	25.7 ± 7.9	15.9	37.5	26.0 ± 7.8	15.4	38.5	24.9 ± 5.4	18.3	34.5
4 (10–14 Feb.)	28.9 ± 7.1	19.3	43.5	27.8 ± 5.1	20.1	37.1	28.4 ± 6.8	19.2	43.2	25.0 ± 3.6	18.8	30.9	27.9 ± 6.5	19.5	36.8
5 (24–28 Feb.)	#	#	#	25.6 ± 2.4	22.6	36.9	24.6 ± 2.5	20.9	31.3	25.4 ± 3.2	20.4	31.8	23.1 ± 2.7	19.6	29.3
6 (17–21 Feb.)	22.8 ± 6.5	13.8	35.1	25.5 ± 7.3	15.3	38.6	23.3 ± 4.8	18.2	34.6	24.9 ± 5.7	17.9	36.8	21.0 ± 2.9	17.9	31.7
7 (17–21 Feb.)	36.7 ± 4.1	29.0	43.1	34.1 ± 9.7	21.1	53.8	26.7 ± 4.1	18.2	32.3	27.9 ± 2.1	24.3	31.0	28.2 ± 1.0	26.2	30.0
8 (3–7 Mar.)	22.6 ± 0.8	21.1	23.9	23.0 ± 0.3	22.2	24.0	23.2 ± 0.4	22.4	24.5	23.6 ± 0.8	22.6	24.9	23.8 ± 0.3	23.4	24.7

Note. # Missing data. No data were recorded on day 1 for School 5; recordings only began on day 2.

STUDY CLIMATE CHANGE, URBAN HEALTH AND VULNERABILITY TO HEAT IN JOHANNESBURG

ASSESSMENTOF HEAT RELATED HEALTH PROBLEMS

GRADE 8 – 12 Pupils

1. Date:____________________
2. Grade:____ Age:___ Sex: ______
3. Name of School:_________________________
4. Do you smoke? No___ or Yes___
5. Do you drink alcohol? No____ or Yes ____
6. Are you physically active (e.g. play any sport); No ____ Yes ____
7. What is your weight? ________
8. What is your height? ________

Please fill the table below. Mark each box under the correct time with an X. First row is for illustration purposes only.

	**Questions**	**Hours**								
		8:00 - 8:59	9:00 - 9:59	10:00 - 10:59	11:00 – 11:59	12:00- 12:59	13:00 - 13:59	14:00- 14:59	15:00 – 15:59	16:00 – 16:59
	Was there a time during the day when you felt tired?			x	x	x				
1	Was there a time during the day when you felt tired?									
2	Was there ever a time during the day when you felt hot than normal?									
3	Was there ever a time during the day when you felt very hot in the head?									
4	Was there ever a time during the day when you lost your concentration?									
5	Was there any time during the day that you felt sick?									
6	Was there any time during the day that you felt dizzy?									
7	Was there any time during the day that you suffered headaches?									
8	Was there any time during school hours that you had nausea?									
9	Was there any time during the day that you had fever?									
10	Was there any time during the day that you felt thirsty than normal (dehydrated)?									
11	Was there a time during the day when you felt sleepy or slept in class?									
12	Was there any time during the day that you developed diarrhoea?									
13	Was there anytime during the day that you were unable to breathe normally?									
15	Was there anytime during the day that you felt slow (i.e. it took you longer to perform tasks than normal)?									

Figure S1. Student questionnaire.

© 2016 by the authors; licensee MDPI, Basel, Switzerland. This article is an open access article distributed under the terms and conditions of the Creative Commons by Attribution (CC-BY) license (http://creativecommons.org/licenses/by/4.0/).
